# Development and Evaluation of an Improved Technique for Pulmonary Function Testing Using Electrical Impedance Pneumography Intended for the Diagnosis of Chronic Obstructive Pulmonary Disease Patients

**DOI:** 10.3390/s131115846

**Published:** 2013-11-21

**Authors:** Myeong Heon Sim, Min Yong Kim, In Cheol Jeong, Sung Bin Park, Suk Joong Yong, Won Ky Kim, Hyung Ro Yoon

**Affiliations:** 1 Biomedical Engineering Research Group, Yonsei University, Wonju 220710, Korea; E-Mails: bmesim@gmail.com (M.H.S.); kmy1207a@naver.com (M.Y.K.); sbpark@yonsei.ac.kr (S.B.P.); wonkykim@yonsei.ac.kr (W.K.K.); 2 Chronic Disease Informatics Program, Johns Hopkins University, Baltimore, MD 21287, USA; E-Mail: decem31@chol.com; 3 Department of Pulmonology, Wonju College of Medicine, Yonsei University, Wonju 220701, Korea; E-Mail: sjyong@yonsei.ac.kr

**Keywords:** spirometry, pulmonary function test, chronic obstructive pulmonary disease, impedance technique, both hands

## Abstract

Spirometry is regarded as the only effective method for detecting pulmonary function test (PFT) indices. In this study, a novel impedance pulmonary function measurement system (IPFS) is developed for directly assessing PFT indices. IPFS can obtain high resolution values and remove motion artifacts through real-time base impedance feedback. Feedback enables the detection of PFT indices using only both hands for convenience. IPFS showed no differences in the sitting, supine, and standing postures during the measurements, indicating that patient posture has no effect on IPFS. Mean distance analysis showed good agreement between the volume and flow signal of IPFS (*p* < 0.05). PFT indices were detected in subjects to differentiate a chronic obstructive pulmonary disease (COPD) patient group from a normal group. The forced vital capacity (FVC), forced expiratory volume in the first second (FEV_1_), FEV_1/_FVC, and peak expiratory flow (PEF) in the COPD group were lower than those in the normal group by IPFS (*p* < 0.05). IPFS is therefore suitable for evaluating pulmonary function in normal and COPD patients. Moreover, IPFS could be useful for periodic monitoring of existing patients diagnosed with obstructive lung disease.

## 1. Introduction

Chronic obstructive pulmonary disease (COPD) is a common and major cause of morbidity, mortality, and airflow obstruction in adults [1–3]. In fact, worldwide, the mortality from COPD and patient has been increasing steadily over the last few decades [4]. Early diagnosis is most effective for managing the progression of COPD, thus, periodic monitoring for prevention has been emphasized. A pulmonary function test (PFT) is useful for the diagnosis, assessment, and management of COPD and other respiratory diseases [5]. For example, in patients already suffering from COPD, regular PFTs can be used to determine whether the condition is worsening [6,7].

Spirometry is the most common type of PFT. It can be categorized into two main types: spirometry used in hospitals (e.g., Vmax Encore from VIASYS Healthcare Inc., Hoechberg, Germany) and Peakflow meters (e.g., Piko-1 from Nspire Health Inc, Longmont, CO, USA, or Vitalography copd-6 from Peal Healthcare Solutions Inc, Delaware, USA) used in home environments. It provides useful diagnosis through measuring and monitoring the lung function of COPD and asthma patients, and for enabling the determination of a therapeutic response to the treatment. The forced vital capacity (FVC) is the maximal volume of air exhaled with maximally forced effort from a maximal inspiration. The forced expiratory volume in the first second (FEV_1_) is the maximal volume of air exhaled in the first second of a forced expiration from a position of full inspiration. FEV_1_/FVC is ratio between FEV_1_ and FVC. The peak expiratory flow (PEF) is the highest flow achieved from a maximum forced expiratory maneuver started without hesitation from a position of maximal lung inflation [8].

The impedance method is a simple technique that requires only the application of two or more electrodes. According to Geddes and Baker, “the impedance between the electrodes reflected seasonal variations, blood flow, cardiac activity, respired volume, bladder, blood and kidney volumes, uterine contractions, nervous activity, the galvanic skin reflex, the volume of blood cells, clotting, blood pressure and salivation” [9]. For these reason, alternative lung function assessment methods have been researched using impedance methods such as impedance pneumography; respiratory inductive plethysmography and various other magnetic, capacitive, and optical methods. For example, electrical impedance pneumography (EIP) indirectly measures the lung functions of patients by sensing the transthoracic electrical impedance variation from the ribcage. The main advantage of EIP is that it can be easily applied; furthermore, it is safe and time-efficient [10]. Goldensohn and Zablow were the first to report a quantitative relationship between the respiration volume and the transthoracic impedance change [11]. The transthoracic impedance voltage change was calibrated with lung volume change, and transthoracic impedance measurements can accurately measure the tidal volume during natural breathing [12,13]. Houtveen *et al.* investigated the transthoracic impedance change and derived respiratory signals obtained from four spot electrodes [14]. Seppa *et al.* investigated the use of EIP for monitoring pulmonary flow and volume signals instead of only the respiration rate or tidal volume [15]. Agarwal *et al.* compared the lung functions of normal, obstructive, and restrictive subject groups by analyzing the frequencies of EIP signals [6]. However, both of these studies could not realize the direct determination of lung function assessment parameters because the impedance signals had limited resolution. Carry *et al.* evaluated the accuracy of EIP [16]. In all these studies, EIP was used to determine various lung function parameters, and the obtained results were compared with those obtained by spirometry. However, these studies which are not concerned about PFT indices (e.g., FEV_1/_FVC, FVC, FEV_1_, and PEF) focused on respiratory rate detection using a belt(s) or attaching an electrode(s) on the patient's chest because of low level respiratory signal resolution [17]. According to the GOLD guidelines, COPD is caused by ventilation, and then COPD can be diagnosed by FEV_1_ and FEV_1_/FVC [18,19].

Therefore, in this study, we developed a hand-held typed impedance pulmonary function system (IPFS) to realize the direct determination of lung function assessment such as the impedance forced vital capacity (IFVC), impedance forced expiratory volume per one second (IFEV_1_), IFEV_1_/IFVC, from volume signals and impedance peak expiration flow (IPEF) from flow signals during respiration. Moreover, the PFT indices obtained by the IPFS can be compared with those obtained by spirometry to enable discrimination between the COPD patient group and the normal group.

## 2. Experimental

### 2.1. IPFS

**Figure 1.**
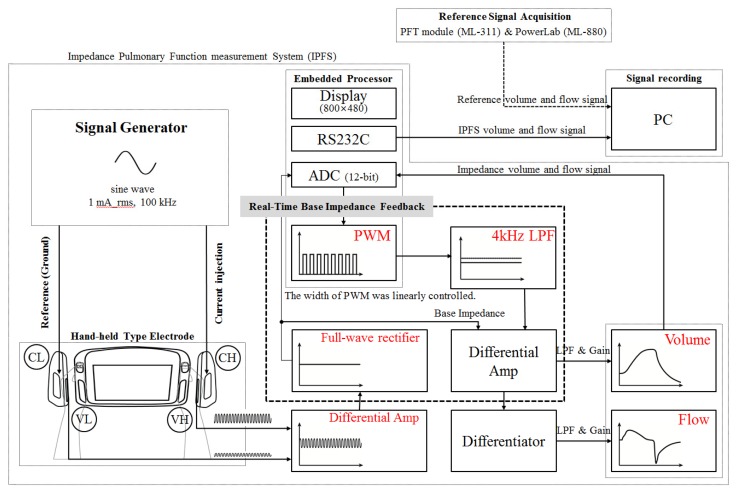
Block diagram of IPFS, CH was used to inject the current and VH and VL were used to measure the voltage generated. CL was used as a reference electrode. Hand-held typed electrodes: electrodes VL and VH (dimensions: 15 × 55 × 2 mm each) and electrodes CL and CH (dimensions: 23 × 55 × 2 mm each). PWM output signals were linearly controlled in accordance with acquired the base impedance.

Figure 1 shows a block diagram of the IPFS. The voltages measured on each hand were differentially amplified. A precision full-wave rectifier was used to acquire the impedance signal without incurring a loss from the differential-amplifier signals. The acquired impedance signal contains the base impedance and the change of impedance during respiration. The total impedance of the right arm, left arm, and thorax are called the “base impedance”.

In previous studies, a high-order high-pass filter for rejection of base impedance was used. In this case, however, the technique removed volume information so as to detect PFT indices when respiration was halted. The width of the pulse width modulation (PWM) was linearly controlled in accordance with the base impedance, as digitized using a Cortex-M3 (32-bit ARM core). The PWM output signal was filtered using a second-order Butterworth low-pass filter with a cutoff frequency of 4 kHz. Therefore, the IPFS can eliminate the base impedance by differentially-amplifying the low-pass filter output signal and the base impedance signal without requiring high-pass filtering. The signal with the eliminated baseline represents the volume; the signal's derivative represents the flow. These volume and flow signals were filtered using a 5 Hz low-pass filter (second-order Butterworth filter) and then amplified. The volume and flow signals were displayed on a 7″ LCD (800 × 480 pixels) and transmitted to a PC after the isolation process. The IPFS used an excitation current of 1 mA (rms) at a frequency of 100 kHz. The current high (CH) electrode was used to inject the current and the voltage high (VH) and voltage low (VL) electrodes were used to measure the voltage generated. The current low (CL) electrode was used as a ground electrode to prevent a floating potential difference, measured by electrodes VH (right hand) and VL (left hand) [20,21]. All electrodes are custom-made, chromium-plated ones.

### 2.2. Signal Processing

**Figure 2.**
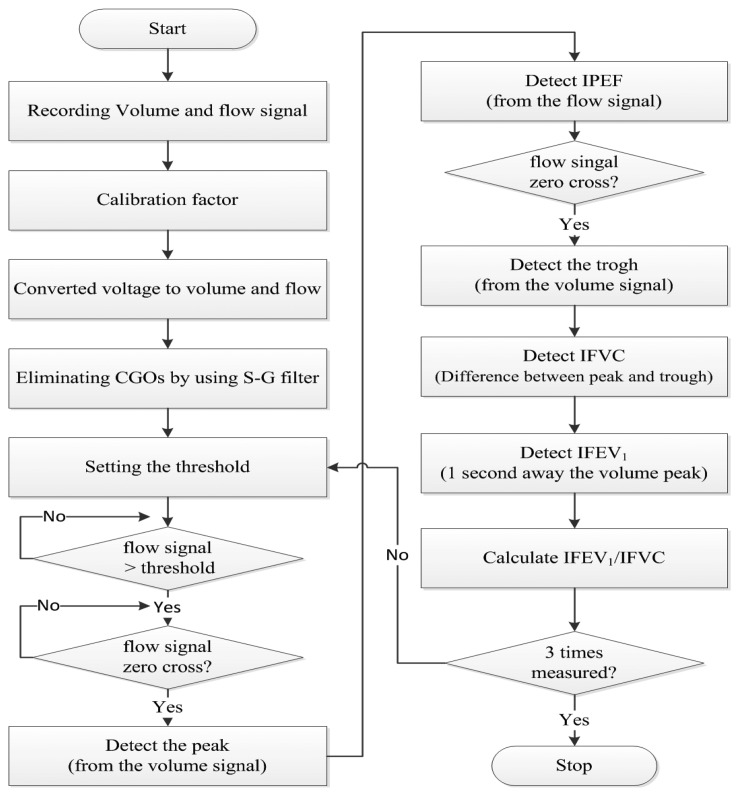
Flowchart for the detection of IPFS indices.

Figure 2 shows a flowchart of the signal processing method. The PFT module's (ML-311; AD Instruments, Bella Vista, Australia) volume and flow signals were recorded on a PC using the PowerLab software (ML-880; AD Instruments). The PFT module and PowerLab are used to acquire reference signals. The IPFS signals were acquired simultaneously. All the signals were sampled at *f*_s_ = 1 kHz. In addition, the voltage-volume and voltage-flow ratios were found using the tidal volume and flow signals in the sitting position of test (1). In this study, we used an ML-311 to calibrate the volume and flow signals of the IPFS. We measured volume and flow raw signals simultaneously, increasing the volume of the spirometry module in four steps (each 100 mL), from 100 mL to 500 mL. The calibration factors (a, b) were calculated using the obtained first-order linear equation for volume (unit: mL) of the spirometry module and the voltage (unit: mV) module of the IPFS. The calibration factors for the IPFS volume were a = 1.260, b = 0.389, and *r* = 0.977, and those of the flow were a = 6.610, b = 1.776, and *r* = 0.955. The output voltage, indicating respiration, is monitored in real-time. The system can generate a constant voltage level using this output voltage. We solved the individual subject calibration problem by generating a personalized constant voltage level using real-time monitoring of the output voltage. Thus, the voltage changes (unit: mV) were converted into to volume (unit: mL) and flow (unit: mL/s) changes via the calibration factor. The IPFS measured the volume signal; the flow signal was determined by differentiating the volume. The measured volume and flow signals are amplified to accurately detect the characteristic points. The IPFS used a Savitzky–Golay smoothing filter (S-G filter) to eliminate the effects of cardiogenic oscillations (CGOs) on the volume and flow signals. This method has the advantage of removing the cardiogenic peak content of the signal [15].

### 2.3. Improvement in Resolution of IPFS

Current is applied to subjects to measure their base impedance, *i.e.*, their body's impedance. Base impedance measurements used a single frequency in the range of 50 to 100 kHz using currents from 0.5 to 4 mA (rms) [22]. The changes in the lung's impedance to airflow (approximately 1 to 10 Ω) are in the range of 0.1% to 1% of the base impedance (approximately 400 to 600 Ω). Therefore, the base impedance is considerably greater than the lung impedance [23]. As shown in Figure 1, a constant current source provides current to the outside electrode CH. The injected constant current (right hand, CH) flows to the ground (left hand, CL). The voltage is measured across Z_Lung_ using a voltage amplifier and electrodes VH and VL.

Assuming the output impedance of the current source is ≫ Z_CH_+Z_Right_arm_+Z_Thoracic_+Z_Lung_+Z_Left_arm_+Z_CL_ and the input impedance of the voltage amplifier is ≫ Z_VH_+Z_Right_arm_+Z_Thoracic_+Z_Lung_+Z_Left_arm_+ Z_VL_, then:

(1) $${\text{Z}}_{\text{Base}+\text{Lung}}={\text{Z}}_{\text{Base}}+\Delta {\text{Z}}_{\text{Lung}}$$

Z_Base+Lung_ has a large steady part, which is proportional to the magnitude of the base impedance Z_Base_, and a small part, ΔZ_Lung_, which represents the change due to respiratory activity.

The output of the voltage differential amplifier is connected to the real-time base impedance feedback system, which removes the base impedance signal, resulting in an improved resolution output voltage, which is proportional to ΔZ_Lung_:

(2) $$\Delta {\text{Z}}_{\text{Lung}}={\text{Z}}_{\text{Base}+\text{Lung}}-{\text{Z}}_{\text{Base}}=({\text{V}}_{\text{Base}}+\Delta {\text{V}}_{\text{Lung}})/\text{I}-{\text{V}}_{\text{Base}}\;/\text{I}=\Delta {\text{V}}_{\text{Lung}}/\;\text{I}$$

**Figure 3.**
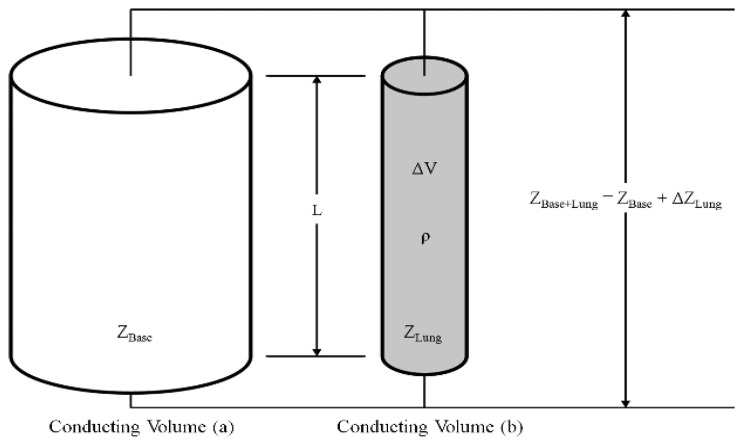
Parallel conducting volumes; the conducting volume (**a**) contains base impedance such right arm, left arm and thoracic and conducting volume (**b**) contains change of the impedance of lung in respiration.

To relate the value of Δ*Z*_Lung_ obtained for the lung to the respiration volume change, a parallel conducting volume model is used [24]. As shown in Figure 3, the conducting volume (a) with impedance *Z*_Base_ consists of a base impedance due to the right arm, left arm, and thoracic impedance. The conducting volume (b) contains the change of lung impedance. The two conducting volumes are (electrically) in parallel. The conducting volume (b) with impedance Δ*Z*_Lung_ has a resistivity ρ, length *L*, and a time-varying cross-sectional area during respiration. Moreover, according to Nopp *et al.* [25], the volume of electrically conductive condensed matter per unit volume of lung tissue is decreased during inspiration. The reduction of electrically conductive condensed matter leads to increasing resistivity of the lung. Therefore, the impedance Δ*Z*_Lung_ is increased. In contrast, the resistivity of the lung and impedance Δ*Z*_Lung_ is decreased during expiration. Accordingly, the impedance change can be expressed in terms of the volume change, as shown in Equations (3) and (4):

(3) $$\Delta {\text{V}}_{\text{Lung}}=\rho ({\text{L}}^{2}/{\text{Z}}_{\text{Base}}^{2})\Delta {\text{Z}}_{\text{Lung}}$$

(4) $$\Delta {\text{V}}_{\text{Lung}}\propto \Delta {\text{Z}}_{\text{Lung}}$$

Δ*V*_Lung_ is the respiration volume change of the lung, ρ is the resistivity of the respiration volume conductor [Ω·cm], *L* is the length of the conducting volume, *Z*_Base_ is the base impedance, and Δ*Z*_Lung_ is the magnitude of the respiration impedance change. The IPFS extracts the volume and flow signals during respiration by measuring the changes in the air inflow impedance during inspiration and expiration. The existing equipment considerably distorts the original signals because the filter removes the base impedance level from the demodulated signal to achieve a high resolution [26]. During inspiration, Δ*V* will increase with Δ*Z*_Lung_, whereas, during expiration, Δ*V* will decrease with Δ*Z*_Lung_. Unlike in spirometry, the proposed IPFS calculates the flow on the basis of changes in volume. The flow is calculated as follows:

(5) $$\text{flow}=\frac{\text{d}}{\text{dt}}{\text{V}}_{\text{Lung}}$$

In particular, the IPFS shows a larger change in the base impedance value due to the body posture because it uses hand-held electrodes. Therefore, the influence of the measurement posture was minimized by removing the initial base impedance value using a real-time base impedance feedback system that uses pulse width modulation. This method determines lung functions by measuring only the impedance from the subjects' hands.

### 2.4. Test Subjects and Measurement Procedure

Thirty subjects participated in this study. They were classified as normal subjects (three males, fourteen females), who had no symptoms of respiratory disease, cardiopulmonary and other diseases or they were classified as COPD subjects (six males, seven females), who had been diagnosed with COPD through a PFT. The clinical procedures were reviewed and approved by the Institutional Review Board of the Wonju College of Medicine, and written informed consent was obtained from all subjects. The following two tests were conducted:

(1) Investigation of effects of subject posture during IPFS measurement.
  The effect of the subjects' postures during IPFS measurement was investigated by measuring natural breaths for 5 min while maintaining each of the following postures: supine, standing, and sitting, in this order. Additionally, to compare the maximal inspiratory and expiratory maneuvers for deriving the PFT indices, forced expiration was conducted in the sitting posture. As shown in Figure 1, the raw volume and flow signals of the PFT module (ML-311; AD Instruments) and the IPFS data were recorded simultaneously.
(2) Comparison of PFT indices obtained using spirometry with those obtained using the IPFS.
  PFTs were conducted on all subjects to compare the PFT indices obtained using spirometry (Vmax Encore; VIASYS Healthcare Inc.) with those obtained using the proposed IPFS. During spirometry, the subjects were instructed to maintain their posture and wear a nose clip. Technicians assisted them during the measurement process. The subjects' tongue and teeth had to be positioned in a certain manner so as not to obstruct the airflow. Each subject wore an airtight seal around their mouthpieces, and performed at least three resting tidal breaths. Then, when instructed, they breathed as deeply and rapidly as possible [8]. If the technicians judged that the PFT results implied that the subjects did not breathe as deeply or rapidly as they could, they repeated the test process to obtain a set of three similar results. Among the results obtained from the repeated measurements, the best results were compared with those obtained using IPFS. The spirometer was calibrated using a 3-L calibration cylinder and was recalibrated before each subject was tested. The room temperature was maintained between 23 and 27 °C.

### 2.5. Statistical Analysis

The data on the influence of measurement positions were presented as *V*_T_ values with interquartile ranges as appropriate. Descriptive data were recorded for all subjects. The parameters' mean values as measured in the three postures were compared using the non-parametric Kruskal-Wallis test [27].

To have a comparison between the reference signals, we also calculated the mean square difference between the normalized signals PFT module and IPFS. As waveforms are normalized, 95% of the values lie mean ±2 SD, then a near maximum value for Δ^2^ is equal to 16 (a situation in which each sample pair is 2 SDs apart) [16]. D is the distance between the IPFS and the spirometry waveform expressed as a percentage of this maximum. The sample of both the volume and the flow signals, *N*_v_ and *N*_f_, respectively, is 7 s:

(6) $$D=100\times \sqrt{\frac{(1/N)\times \overset{N}{\underset{k=1}{\text{∑}}}{\left[V_{\text{PFT}}(k)-V_{\text{IPFS}}(k)\right]}^{2}}{\Delta^{2}}}k=1\;to\;N,N=7000$$

To validate the use of IPFS with COPD patients, the correlations of the indices were evaluated by a simple regression and Bland-Altman analysis between spirometry and IPFS[28]. The statistical programs used in this study were SPSS 17.0 (SPSS Inc., Chicago, IL, USA) and MedCalc 12.0 for Windows (MedCalc Software, Ostend, Belgium).

## 3. Results and Discussion

### 3.1. Measurement Results by Posture

To eliminate the influence of posture on the IPFS, tidal volume changes were measured for each posture and each group. Table 1 presents a comparison of the *V*_T_ values obtained using IPFS at each posture for all subjects. The detection error of the PFT indices can be minimized for all measurement positions of *V*_T_; the error ratios in the supine position and standing position were less than 5%. The tidal volume for 30 subjects (COPD patient: *n* = 13, normal group: *n* = 17) was estimated in sitting, supine, standing positions, respectively, to validate the base impedance rejection performance. The error in Table 1 refers to how much greater or less tidal volume is estimated when subjects were in supine and standing position on the basis of sitting position.

Consequently, we obtained a mean error of 1.52% and a standard deviation of −2.10% to 16.75% in the supine position; and a mean error of 1.59% and a standard deviation of −3.73% to 3.75% in the standing position. From the above results, we can be sure that there is no statistically significant difference in tidal volume for the sitting, supine, and standing postures, because the significance level (*p*-value = 0.991) of the nonparametric Kruskal-Wallis test is greater than 0.05. Therefore, the IPFS confirmed that the removal of both the base impedance of the human body (e.g., left arm, right arm, and thorax) and the impedance caused by the initial measurement posture improved performance.

### 3.2. Agreement between Volume and Flow Signals for PFT and IPFS

**Figure 4.**
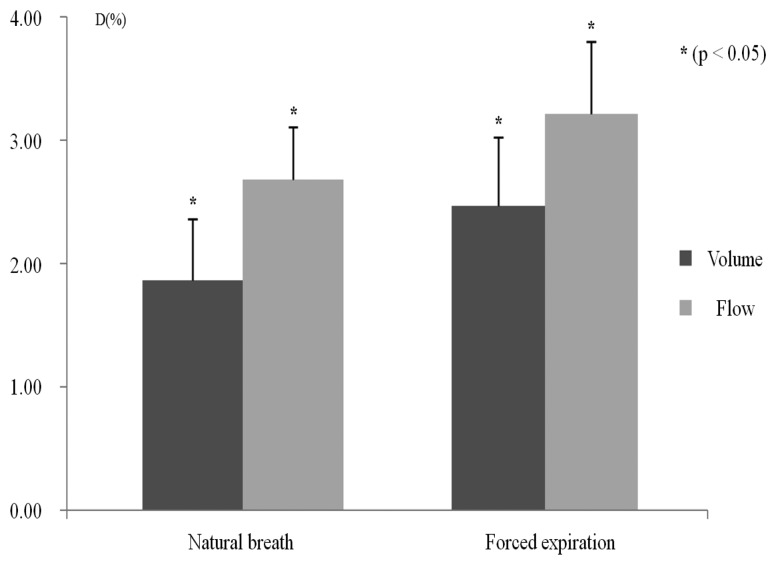
Mean distance between natural breath (*V*_T_) and forced expiration (PFT) for all subjects.

The agreement between the volume and the flow signals was evaluated for all subjects without separating them into groups. Figure 4 shows the agreement between natural breaths and forced expiration for the PFT module, and the IPFS's volume and flow signals. To compare the waveforms of these two signals, we eliminated any discrepancy due to baseline or amplitude differences. These conditions were realized by normalizing each signal waveform. Statistical analysis shows that the mean distances of the volume and flow of natural breath are significantly lower than those of the volume and flow of forced expiration (*p* < 0.05). In both natural breath and forced expiration, the flow signals had larger errors than the volume signals. For this reason, the effects of CGOs on the volume signals were eliminated, whereas those of flow signals were not completely eliminated because of the differentiator's characteristics. However, the flow signal error rate due to the differentiators was approximately 0.6%. Moreover, the PEFs of the flow signals are not strongly influential when identifying COPD patients from all subjects.

### 3.3. Classification Function between Normal and COPD Patient Group

**Figure 5.**
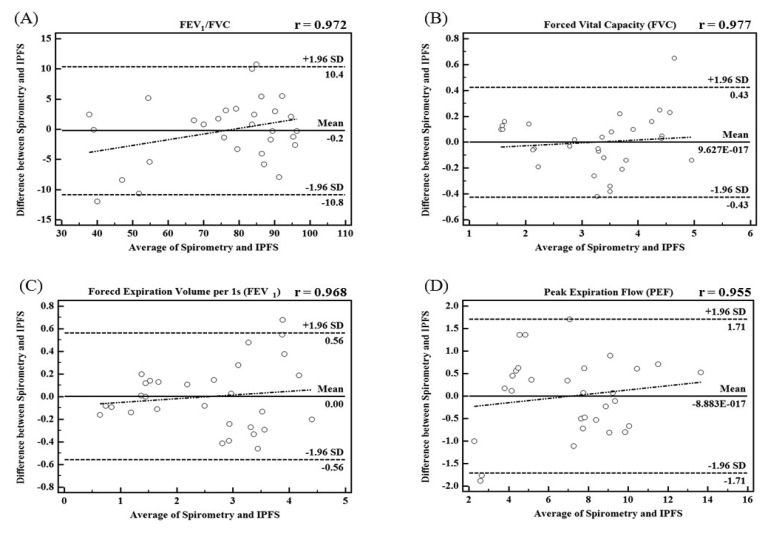
Bland–Altman plots about PFT indices between IPFS and spirometry; (**a**) FEV_1_/FVC; (**b**) FVC; (**c**) FEV_1_; (**d**) PEF.

**Figure 6.**
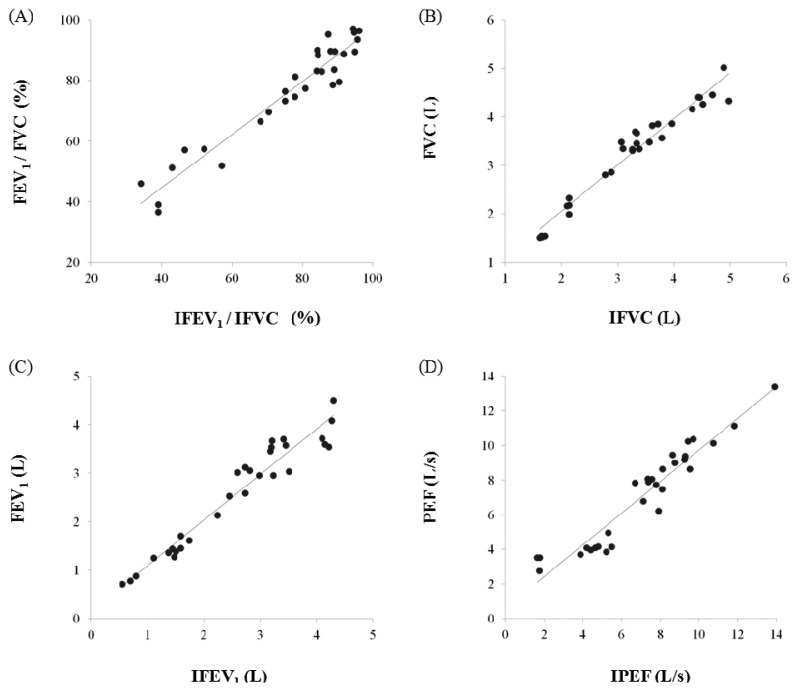
Correlation between PFT and IPFS indices: (**a**) FEV_1_/FVC; (**b**) FVC; (**c**) FEV_1_; (**d**) PEF.

In this study, we proposed the IPFS for improving upon the disadvantages of existing spirometric and impedance-based methods for the determination of lung function assessment parameters such as FEV_1_/FVC, FVC, FEV_1_, and PEF. To validate the performance of the IPFS, the indices of the IPFS were evaluated using a simple regression and Bland-Altman analysis. The flow signals were determined using volume as the criterion; the IPEF was found to be lower than the other indices (IFEV_1_/IFVC, IFVC, and IFEV_1_). The correlation values were obtained using the IPFS: IFEV_1_/IFVC (*r* = *0.972*), IFVC (*r* = *0*.*977*), IFEV_1_ (*r* = *0.968*), and IPEF (*r* = *0.955*) (*p* < *0*.*05*). IFVC, IFEV_1_, and IFEV_1_/IFVC are the volume indices of PFT. IPEF is the flow index of PFT. PFT indices obtained by spirometry were well correlated with those obtained by the IPFS for all subjects (*p* < *0*.*05*). Figure 5 shows the plots of the average values for IPFS and spirometry (x-axis) and the differences between IPFS and spirometry values (y-axis). The Bland–Altman plots indicate that 95% of the mean differences fell within the limits of agreement for FEV_1_/FVC (Figure 5a), FVC (Figure 5b), FEV_1_ (Figure 5c), and PEF (Figure 5d). In addition, the Bland–Altman plot's regression lines show that as the average values obtained by spirometry and the IPFS increased, so did the differences between the values obtained by spirometry and IPFS. These results imply that the PFT indices for all subjects (*n* = 30) are widely distributed, and therefore, the values estimated using the proposed IPFS are statistically reliable. Figure 6 shows the correlations between the PFT index measurements for both IPFS and spirometry. The PFT indices are FVC, FEV_1_, and FEV_1_% for the volume and PEF for the flow signal. PFT indices obtained using spirometry (x-axis) were well correlated with those obtained using the IPFS (y-axis) when analyzed for all subjects (*p* < *0*.*05*).

Using the PFT indices measured by the IPFS, the subjects were classified into normal and COPD patient groups. These PFT indices were compared with those obtained using spirometry. Table 2 shows the results of individual patient classification of PFT indices, which were measured through IPFS and spirometry. The classification was made according to the COPD gold standard [19]. The COPD assessment criteria are as follows: FEV_1_ < 80% of the predicted value in combination with FEV_1_/FVC < 70% confirms the presence of airflow limitation [22]. The classification results of all subjects were identical, except for subject 9. The statistical significance was confirmed in the classification of normal and COPD patient groups using the developed IPFS because the classified results had an accuracy of 92% detection ratio.

## 4. Conclusions

The proposed IPFS can be used to determine lung function assessment parameters such as the FVC, FEV_1_, and FEV_1_/FVC from volume signals and PEF from flow signals, and these indices can enable classification between normal and COPD patient groups. Unlike spirometry, the IPFS determined the signal flow from the volume. Previous studies on detecting respiration changes using the impedance method [6,15] did not report the parameters used for the assessment of lung function because impedance signals involving respiration had limited resolution. In addition, the attachment of electrodes to the chest inconveniences the patient.

IPFS is more convenient than the conventional method for users, because the IPFS measures the volume and flow of breathing using only the hands. The base impedance values for both-hands, arms, and chest were removed using a real-time base impedance feedback system. The variation in the amount of respiratory impedance is less than the change in the base impedance's value. Therefore, it is important that the base impedance has been completely removed and then amplified to improve the accuracy of the respiratory characteristic point detection ratio. This problem is easily shown for the system at low voltage operation. On the other hand, low voltage (e.g., a single 3.3 V system) operation system such as the IPFS can solve this problem using real-time base impedance feedback. Moreover, this system enables the calculation of the tidal volume and the PFT indices through the detection of the respiration signals.

The subjects were classified into two groups, the COPD patient group and the normal group, using the PFT indices measured by the IPFS. These PFT indices were compared with those obtained using spirometry. The COPD assessment criteria to confirm the presence of airflow limitation are as follows: FEV_1_ < 80% of the predicted value and FEV_1_/FVC < 70% [22]. As shown in Table 2, the PFT indices for all subjects were determined using IPFS. All subjects were classified into normal (subjects 1–5, 19–30) or COPD patient groups (subjects 6–18) by the specialist during the experiment. It is possible to classify the normal and COPD patient groups using this result. A spirometric curve of lung volume and the FEV_1_ parameter of the force expiratory volume curve have been used as important clinical indicators of the distinction between obstructive lung disease and restrictive lung disease.

Arterial blood gas (ABG) measurement is important clinical test that represents indicators for a comprehensive evaluation of two parts of respiration, the degree of ventilation and perfusion. However, the progress of lung disease (e.g., COPD, asthma) can make a decision by monitoring of respiratory volume and flow because ventilation is more problematic than perfusion for these diseases. In the GOLD guidelines, it has been mentioned about the COPD assessment criteria [19]. In the GINA guidelines, the most important use in the diagnosis and treatment of asthma, PEF is used as a significant indicator of asthma and PEF value is capable by comparison of own previous best measurement [18]. The IPFS using electrical impedance pneumography method can be detected the PFT indices (FEV_1_/FVC, FVC, FEV_1_, and PEF) conveniently. IPFS only use hand-held typed electrodes, not use ancillary equipment. Especially, IPFS is able to estimate COPD indices without any experienced technicians. Therefore, IPFS could be useful for periodic monitoring of patients diagnosed with obstructive lung disease. For those reasons, our study is about a novel system capable of pulmonary function indices detection for COPD prevention and diagnosis through impedance technique.

Our study was limited to a small sample size and studied only normal and COPD patient groups. In future studies, when the volume-flow curve is suggested for detecting the exact characteristics of the ventilation pattern, it can be used for examining pulmonary disease as well as the classifying normal and COPD group by using IPFS.

## Figures and Tables

**Table 1.** Comparison of tidal volume (*V*_T_) values in each posture of IPFS.

**Subject**	**A(1)**	**b(2)**	**c(3)**	**d(4)**	**e(5)**
1	560.99	566.32	0.95	563.53	0.45
2	510.54	511.84	0.26	507.90	0.52
3	478.72	483.93	1.09	486.38	1.60
4	494.63	493.52	0.23	492.60	0.41
5	374.18	374.73	0.15	379.18	1.34
6	242.36	244.06	0.70	237.83	1.87
7	301.45	300.73	0.24	308.30	2.27
8	311.00	313.99	0.96	318.51	2.41
9	274.18	320.10	1.49	270.10	2.06
10	257.36	265.26	3.07	262.05	1.82
11	220.82	229.27	3.83	229.09	3.75
12	214.18	215.03	0.40	212.82	0.63
13	242.36	238.32	1.67	233.56	3.63
14	235.54	230.60	2.10	239.23	1.57
15	255.68	255.79	0.04	246.14	3.73
16	358.27	367.20	2.49	367.64	2.61
17	253.16	251.74	0.56	247.14	2.38
18	274.18	280.63	2.35	275.98	0.66
19	478.72	475.72	0.63	483.34	0.97
20	529.17	526.01	0.60	529.25	0.01
21	412.36	408.90	0.84	407.39	1.20
22	503.95	501.07	0.57	508.87	0.98
23	415.72	413.38	0.56	406.43	2.23
24	475.36	474.39	0.20	474.87	0.10
25	445.08	439.13	1.34	456.17	2.49
26	408.86	412.20	0.82	417.98	2.23
27	434.09	438.95	1.12	429.10	1.15
28	466.04	466.05	0.00	467.97	0.41
29	381.18	384.93	0.99	379.06	0.56
30	414.81	415.56	0.18	405.45	2.26

(*p*-value = 0.991) is significance level of the nonparametric Kruskal-Wallis test according to each posture. *V*_T_ = Tidal volume (mL);

(1) Sitting *V*_T_ (mL);

(2) Supine *V*_T_ (mL);

(3) Error of supine (%). The *V*_T_ error ratio of supine against sitting posture;

(4) Standing *V*_T_ (mL);

(5) Error of standing (%). The *V*_T_ error ratio of standing against sitting posture.

**Table 2.** Result of PFT indices for normal group and COPD patents.

**Subject**	**PEF (L/s)**		**FVC (L)**		**FEV_1_/FVC (%)**		**FEV_1_(FEV_1_% Predicted)(L)**			**Classification**

	**Spirometry**	**IPFS**	**Spirometry**	**IPFS**	**Spirometry**	**IPFS**	**Spirometry**	**IPFS**	**Predicted**	
1^i^	11.84	11.13	4.88	5.02	88.11	89.87	4.30(102.63)	4.51(107.58)	4.19	Normal
2^i^	13.91	13.38	4.68	4.45	90.17	79.58	4.22(104.71)	3.54(87.87)	4.03	Normal
3^i^	7.39	7.89	3.31	3.69	96.07	93.57	3.18(103.92)	3.46(112.92)	3.06	Normal
4^i^	9.71	10.37	3.71	3.85	86.52	95.33	3.21(99.38)	3.67(113.67)	3.23	Normal
5^i^	8.65	9.46	3.26	3.33	91.41	88.61	2.98(108.36)	2.95(107.25)	2.75	Normal
6^i^	5.31	4.95	4.45	4.40	39.10	36.29	1.74(83.25)	1.60(76.35)	2.09	COPD
7^ii^	5.22	3.86	2.77	2.80	57.40	51.38	1.59(71.62)	1.44(64.81)	2.22	COPD
8^ii^	5.50	4.14	2.13	2.32	65.26	67.16	1.39(68.81)	1.56(77.24)	2.02	COPD
9iii	7.12	6.77	2.88	2.86	67.71	74.36	1.95(53.93)	2.13(59.88)	3.55	Normal
10^ii^	4.65	4.09	2.13	1.99	70.42	68.92	1.50(86.71)	1.37(79.30)	1.73	COPD
11^ii^	1.64	3.52	1.61	1.51	42.86	50.07	0.69(46.31)	0.76(50.72)	1.49	COPD
12^ii^	1.77	3.53	1.70	1.54	46.47	56.00	0.79(52.32)	0.86(57.09)	1.51	COPD
13^ii^	4.20	4.08	3.56	3.48	39.48	38.70	1.37(56.61)	1.35(55.59)	2.42	COPD
14^ii^	3.87	3.70	2.13	2.18	52.11	56.77	1.11(67.27)	1.24(74.94)	1.65	COPD
15^ii^	4.41	3.96	2.10	2.16	68.57	65.89	1.44(68.57)	1.42(67.79)	2.10	COPD
16^ii^	7.92	6.21	3.26	3.31	69.02	68.63	2.25(78.67)	2.27(79.33)	2.86	COPD
17^ii^	1.76	2.76	1.64	1.54	33.54	44.79	0.55(48.67)	0.69(61.17)	1.13	COPD
18^ii^	4.79	4.16	1.65	1.52	64.85	66.01	1.07(62.57)	1.00(58.70)	1.71	COPD
19^i^	7.78	7.71	4.97	4.32	84.50	83.21	4.15(90.22)	3.60(78.22)	4.60	Normal
20^i^	9.53	8.63	4.32	4.16	94.91	89.49	4.10(122.02)	3.72(110.70)	3.36	Normal
21^i^	8.09	7.47	3.33	3.67	96.10	96.42	3.20(109.59)	3.53(121.06)	2.92	Normal
22^i^	10.74	10.13	4.51	4.26	94.68	96.03	4.27(129.00)	4.09(123.50)	3.31	Normal
23^i^	8.76	8.99	3.96	3.86	88.64	78.56	3.51(117.00)	3.03(100.97)	3.00	Normal
24^i^	9.28	9.39	4.43	4.40	77.88	81.23	3.45(107.48)	3.58(111.46)	3.21	Normal
25^i^	7.57	8.04	3.09	3.35	84.14	89.91	2.60(83.87)	3.01(97.06)	3.10	Normal
26^i^	9.25	9.18	3.78	3.56	85.45	82.93	3.23(119.19)	2.95(108.97)	2.71	Normal
27^i^	6.71	7.82	3.06	3.48	89.22	89.50	2.73(106.23)	3.11(121.21)	2.57	Normal
28^i^	8.11	8.64	3.38	3.34	80.77	77.25	2.73(95.12)	2.58(89.89)	2.87	Normal
29^i^	7.35	8.07	3.33	3.45	84.38	88.36	2.81(98.94)	3.05(107.37)	2.84	Normal
30^i^	9.44	10.24	3.61	3.82	94.46	97.11	3.41(107.91)	3.71(117.27)	3.16	Normal

PEF = peak expiratory flow, FVC = forced vital capacity, FEV_1_/FVC = ratio of FEV_1_/FVC, FEV_1_(FEV_1_% Predicted) = forced expiratory volume in 1 s (ratio of FEV_1_/FEV_1_ Predicted). Classified result from FEV1/FVC of IPFS and FEV1% Predicted in Normal group and COPD patient group (Classification): Subject

i The case distinguished Normal group from Normal group. Subject

ii The case distinguished COPD group from COPD group. Subject

iii The case distinguished Normal group from COPD group.
